# Comparison of the effectiveness and safety of intravenous and topical regimens of tranexamic acid in complex tibial plateau fracture: a retrospective study

**DOI:** 10.1186/s12891-020-03772-7

**Published:** 2020-11-12

**Authors:** Zhimeng Wang, Yao Lu, Qian Wang, Leilei Song, Teng Ma, Cheng Ren, Zhong Li, Jiarui Yang, Kun Zhang, Bing Zhang

**Affiliations:** 1 Department of Orthopaedics and Trauma, Hong Hui Hospital, Xi’an Jiaotong University College of Medicine, No. 555, East Youyi Road, Xi’an, 710000 Shaanxi China; 2 Department of Orthopaedics and Trauma, The Second Affiliated Hospital of Xi’an Medical College, No. 167, East Textile Road, Xi’an, 710000 Shaanxi China; 3 The Key Laboratory of Biomedical Information Engineering of Ministry of Education, School of Life Science and Technology, Xi’an Jiaotong University, Xi’an, 710049 People’s Republic of China; 4 Bioinspired Engineering and Biomechanics Center (BEBC), Xi’an Jiaotong University, Xi’an, 710049 China; 5 Qinghai University, Xi’ning, 810000 Qinghai China

**Keywords:** Tranexamic acid, Hyperfibrinolysis, Tibial plateau fracture

## Abstract

**Background:**

Previous studies have demonstrated the effectiveness and safety of tranexamic acid (TXA) in orthopedic surgery. However, no study has investigated TXA in complex tibial plateau fracture surgery. Therefore, the purpose of this study was to confirm the safety and effectiveness of i.v. (intravenous) TXA and topical TXA.

**Material and methods:**

This was a retrospective analysis of prospectively collected data. The control group received an equal amount of placebo (physiological saline solution); the i.v. group received 1.0 g TXA by intravenous injection before the tourniquet was inflated and before the surgical incision was closed, and the topical group received 3.0 g TXA in 75 mL of physiological saline solution 5 min prior to the final tourniquet release. Perioperative blood loss, vascular events, wound complications, and adverse reactions were compared among the three groups. The pain, knee function, and quality of life (QoL) assessments were based on their corresponding scoring systems.

**Results:**

Baseline data were comparable for all groups. The i.v. group showed the best results for total blood loss (TBL) and hidden blood loss (HBL) (424.5 ± 49.4 mL and 219.3 ± 33.4 mL, respectively, all *P* values < 0.001). Patients in the i.v. group had lesser real Hb decrease than those in the control group (0.9 vs 1.5, *P*<0.001) and topical group (0.9 vs 1.2, *P* = 0.026). The blood coagulation level as measured using fibrinolysis (D-dimer) was lower in the i.v. group than in the control and topical groups on POD1 and POD3; however, this difference was not significant; the fibrin-degradation products also showed a similar trend. Patients in the topical group experienced less pain than those in the control group on POD2, POD4, and PO6W. The VAS pain score was 3.6 vs. 4.4 (POD2, *P*<0.05), 2.8 vs 3.3 (POD4, *P*<0.05), and 2.1 vs. 2.6 (PO6W, *P*<0.001) in the topical group vs control group, respectively. No significant differences were identified in vascular events, wound complications, adverse reactions, knee function, and QoL among the three groups.

**Conclusion:**

To our knowledge, this is the first study that showed both i.v. TXA and topical TXA are safe and effective for complex tibial plateau fractures. The i.v. regimen effectively reduced blood loss during the perioperative period, whereas patients under the topical regimen had less vascular events, wound complications, and a lower incidence of adverse reactions compared to those in the i.v. group.

**Trial registration:**

The trial was registered in the Chinese Clinical Trial Registry (ChiCTR-TRC-1800017754, retrospectively registered from 2018 to 01-01).

## Background

Tibial plateau fractures are mostly caused by high-energy direct impact; however, the incidence of osteoporotic fracture of the tibial plateau increases with age [1, 2]. For this type of fracture, surgical treatment can improve the quality of life (QoL) of patients as well as their knee function [3]. Most simple types of tibial plateau fractures (Schatzker I–III) are not difficult to treat. Owing to the local anatomic characteristics of the posterior and lateral sides of the tibial plateau, exposure and fixation of complex tibial plateau fractures (Schatzker V and VI) represent major challenges [4]. Schatzker type V and VI tibial plateau fractures are usually caused by severe crushing and collapse of the medial and lateral condyles, usually owing to high-energy trauma. The use of posteromedial and anterolateral incisions combined with bone grafting and double plate fixation can maintain the absolute stability of the tibial plateau while also satisfying the biological effects of the bone and surrounding soft tissues [5]. Since there is an abundance of blood vessels around the knee joint, an increase in the incision exposure range and the use of tourniquets can lead to profuse blood loss and fibrinolytic response during the perioperative period [6]. Antifibrinolytic therapy, an important aspect of blood management that is considered to be closely related to the concept of enhanced recovery after surgery (ERAS), has emerged as a particular research focus [7, 8].

Tranexamic acid (TXA) is a traditional antifibrinolytic drug that binds to lysine residues and forms a reversible complex with plasminogen and plasmin. It is an effective agent against plasmin, tissue plasminogen activators, and plasminogen [9]. Several studies have reported that TXA can effectively reduce the rate of perioperative blood transfusion, blood loss, and drainage volume without increasing the risk of deep vein thrombosis (DVT) formation in trauma orthopedics [10–14]. In addition, it has been shown to be cost-effective.

Currently, TXA is administered as intravenous, intramuscular, topical, and oral regimens. Since the safety of intramuscular and oral regimens has not been supported by a large number of clinical trials, these routes of administration were excluded from our study. Some scholars have suggested that when TXA is used topically in the joint cavity, the drainage must not be placed after surgery to avoid loss of drug efficacy [15, 16]. Therefore, to reduce bias in test results, a temporary clamping scheme for the drainage tube after the operation was used in this study [17].

Given the encouraging results for TXA, we designed this study to evaluate the efficacy and safety of the intravenous and topical use of TXA in Schatzker V and VI tibial plateau fractures. We hypothesized that, compared to the control group, both intravenous TXA and topical TXA regimens can reduce blood loss and facilitate postoperative recovery without increasing thromboembolic complications.

## Materials and methods

The present study is a part of a randomized controlled trial (RCT), which is registered in the Chinese Clinical Trial Registry (ChiCTR-TRC-1800017754); thus, the reported study is a retrospective analysis of prospectively collected data. Approval was obtained from the Clinical Trials and Biomedical Ethics Committee of Hong Hui Hospital (Approval Number: 2018002), and written informed consent was obtained from all participants.

### Patients

Ninety patients with diagnoses of Schatzker V and VI tibial plateau fractures from January 2018 to February 2020 were recruited. The inclusion criteria were as follows: (1) patients more than 18 years of age; (2) patients showing the presence of a unilateral closed tibial plateau fracture with an image inspection that conformed to Schatzker V and VI classification standards; (3) patients showing no coagulopathy or abnormal hemoglobin before operation; and (4) fresh fracture, with a period between injury to hospital admission of less than 3 days. The exclusion criteria were as follows: (1) patients with severe brain, heart, liver, and kidney dysfunction, who could not tolerate surgery; (2) patients with coagulation dysfunction; (3) patients with pathological fractures or tumors; (4) patients with bilateral tibial plateau fractures or other injuries; (5) patients in whom TXA or anticoagulant drugs were contraindicated; (6) patients with incomplete data; (7) preoperative ultrasonography that shows DVT; and (8) the use of spanning external fixation as a primary treatment upon patient admission. In addition, if the surgeon chose to fill the collapsed articular surface with an autologous bone graft during surgery, that patient’s data were excluded from statistical analysis, as it was considered that the extra wound incision and blood loss from the iliac bone may have affected the results.

### Intervention

Patients were divided into the control group, i.v. group, and topical group based on a computer-generated randomization schedule. The control group received an equal amount of placebo (physiological saline solution) as i.v. (total 200 mL) and topical (75 mL) treatments. The i.v. group received 1.0 g i.v. TXA before the tourniquet was inflated and before the surgical incision was closed. Five minutes prior to the final tourniquet release, the topical group received 3.0 g TXA in 75 mL of physiological saline solution, which was injected retrogradely into the joint cavity through a drainage tube placed deep in the incision. The drainage tube was temporarily clamped for 4 h after the operation. In the control group, an equal volume of saline was used. The therapeutic doses of these regimens were determined from previous studies [17–20].

### Surgical methods and postoperative management

All other drugs, except for TXA and physiological saline solution during general anesthesia, were the same in all three groups. The pressure of the balloon-type tourniquet on the affected limb was set to 450 mmHg, and the mean arterial blood pressure (MAP) was maintained at 60–70 mmHg. Surgical procedure as described previously [5, 21]. Briefly, a medial and lateral double incision approach combined with double locking steel plates was used to fix the fracture block. Primarily, a medial incision (posterior medial approach) was performed to expose the fractured end of the medial side. Kirschner wire was used for temporary reduction and this area was fixed with the anatomical locking plate of the proximal tibia. Then, the joint was opened using a lateral incision (anterolateral approach) to fully expose the lateral condyle; the lateral dissection plate of the proximal tibia was used for fixation and artificial bone grafts were used. Before the incision was closed, two drainage tubes were placed in deep part, where the incision was sutured layer by layer. After compressing the sutured incision with a self-adhesive elastic bandage, the tourniquet was loosened. The drainage pipes for each patient were temporarily clamped for 4 h after the operation. After the operation, the leg was raised for 3 to 4 days, and the drainage tubes were removed within 48 h. Functional exercises related to the active and passive ranges of motion were started approximately 2 weeks after surgery. The RBC transfusion indications formulated by the Chinese Ministry of Health are as follows: 1) Hb < 70 g/L, and 2) 70 g/L < Hb < 100 g/L, when the patient has symptoms of dizziness, palpitation, asthma, and fatigue.

### Study objectives

The following demographic data were recorded for each patient: sex, age, body mass index (BMI), medical history, American Society of Anesthesiologists score [22] (ASA), fracture type, and preoperative levels of hemoglobin (Hb), hematocrit (Hct), D-dimer, and fibrinogen (FIB). The operation time and intraoperative blood loss were also included in the statistics. Furthermore, laboratory data (e.g., Hb, Hct, D-dimer, and FIB) were also evaluated 24 h after surgery and on the third postoperative day (POD 3). To evaluate the safety of TXA in this study, any vascular event that occurred within 12 weeks after surgery was assessed, including DVT of the lower extremity (confirmed using ultrasound), pulmonary embolism (PE, confirmed using pulmonary spiral CT), cerebrovascular accidents (confirmed using spiral CT or MRI), gastrointestinal hemorrhage, as well as the incidence of wound complications (e.g., dehiscence, hematoma, edge necrosis, and infection). Potential adverse side effects of TXA, including epilepsy, rash, headache, nausea, and vomiting, were also monitored. The Hospital for Special Surgery (HSS) knee score [23], a 12-item short form health survey (SF-12) [24] and visual analog scale (VAS) [25, 26] were used to evaluate patient outcomes. Owing to the granularity of the SF-12 scoring system, we divided it into two parts, namely, the physical component summary (PCS) and mental component summary (MCS), for comparison.

### Calculation of perioperative blood loss

Perioperative total blood loss (TBL) and hidden blood loss (HBL) were the focal points of this study. Intraoperative blood loss (IBL) was estimated from the weight of the surgical sponges and the measurement of the volume of blood collected by the suction canisters. The weight of the irrigation fluids added to the surgical field and the sponge weight were then subtracted from this value. The total postoperative drainage (TPD) was the weight of fluid in the drainage bag collected over 48 h.

Primarily, the preoperative blood volume (PBV) was calculated using Nadler’s equation [27]: PBV (L) = K_1_ × h^3^ + K_2_ × w + K_3_, [h: height (m), w: weight (kg)]; for male patients, K_1_ = 0.3669, K_2_ = 0.03219, and K_3_ = 0.6041; for female patients, K_1_ = 0.3561, K_2_ = 0.03308, and K_3_ = 0.1833. The total blood loss (TBL) was calculated according to the Gross formula [28]: TBL (mL) = PBV × (Hct_1_ – Hct_2_) + Hb_trans_, where Hct_1_ is the first routine blood test after the patient was admitted to the hospital, Hct_2_ is the lowest value obtained by routine blood tests after surgery (24 h or POD3), and Hb_trans_ is the weight of the transfused packed red blood cells (PRBCs), where two units of PRBCs can cause an Hb increase of approximately 5.2 g/dL, with a volume of approximately 400 mL [29]. All transfused blood products were fresh and stored frozen.

Lastly, hidden blood loss (HBL) was calculated as follows:

$$ \mathrm{HBL}\ \left(\mathrm{mL}\right)=\mathrm{TBL}\hbox{-} \mathrm{IBL}\hbox{-} \mathrm{TPD} $$

### Statistical analysis

Statistical analyses were performed using GraphPad Prism 8.0. Continuous variables were reported as the mean and standard deviation. One-way analysis of variance (ANOVA) was used to compare the differences among multiple groups. Student’s *t*-test was used to compare the difference between two groups and the Chi-square test was used for the analysis of categorical data. *P* values < 0.05 were considered to indicate a statistically significant difference.

## Results

### Patient demographics

All 90 patients included in the study were followed up postoperatively for 12 weeks, so no patients were lost to follow-up. There were no significant differences in the demographic data and preoperative blood test results among the three groups of patients (Table 1).

**Table 1:** Patient demographic data and preoperative blood test results

Variable	Control group (30 patients)	i.v. group (30 patients)	Topical group (30 patients)	*P* value (between groups)
Patient characteristics				
Age (yr)	44.6 ± 6.7	43.8 ± 5.5	45.1 ± 6.3	0.715^a^
Gender (male/female)	24/6	25/5	28/2	0.311^b^
BMI	23.1 ± 1.4	22.9 ± 1.5	23.2 ± 1.7	0.745^a^
Medical history				
Diabetes mellitus	1	1	1	1.000^b^
Hypertension	3	1	2	0.585^b^
Arrhythmia	1	2	2	0.809^b^
ASA score				
I	24	24	23	0.966^b^
II	4	5	5	
III	2	1	2	
Schatzker type				
V	14	17	11	0.300^b^
VI	16	13	19	
Preoperative blood tests				
Hb (g/dL)	12.7 ± 1.0	12.8 ± 0.9	13.1 ± 1.4	0.360^a^
Hct (%)	39.2 ± 2.7	39.1 ± 2.1	40.1 ± 3.1	0.283 ^a^
D-dimer (mg/L)	6.3 ± 1.2	6.7 ± 1.7	6.1 ± 0.8	0.191 ^a^
FIB (g/L)	4.6 ± 1.1	5.2 ± 1.5	4.8 ± 1.2	0.172 ^a^

*Abbreviations*: *BMI* Body mass index, *ASA* American Society of Anesthesiologists, *Hb* Hemoglobin, *Hct* Hematocrit, *FIB* fibrinogen

Intergroup comparisons performed using ANOVA or Chi-square test (^a^ANOVA; ^b^Chi-square test)

### Operation time, IBL, and TPD

The mean operation time in the control, i.v., and topical groups was 117.7 ± 19.4, 109.1 ± 17.5, and 115.3 ± 20.5 min, respectively. There was no significant difference between these values (*P* = 0.206), although the time in the i.v. group was shorter than in the other groups. The average values of IBL in the control, i.v., and topical groups were 144.5 ± 21.1, 116.5 ± 15.2, and 137.5 ± 19.2 mL, respectively, which were significantly different (*P* <  0.001); the best effect occurred in the i.v. group (P <  0.001). The topical group showed the greatest reduction in TPD among the three groups, with a significant difference compared to the i.v. group (64.1 mL vs 76.5 mL, *P* = 0.004) (Table 2).

**Table 2:** Postoperative data and postoperative blood test results

Variable	Control group (30 patients)	i.v. group (30 patients)	Topical group (30 patients)	*P* value (between groups)	Intergroup comparison		
					*P*_*1*_ *P*_*2*_ *P*_*3*_		
Surgical data							
Duration of surgery (min)	117.7 ± 19.4	109.1 ± 17.5	115.3 ± 20.5	0.206^a^	–	–	–
Duration of tourniquet (min)	97.3 ± 8.5	95.4 ± 10.1	101.6 ± 11.4	0.056^a^	–	–	–
IBL (mL)	144.5 ± 21.1	116.5 ± 15.2	137.5 ± 19.2	< 0.001^a^	< 0.001	0.184	< 0.001
TPD (mL)	156.3 ± 27.6	76.5 ± 15.2	64.1 ± 16.3	< 0.001^a^	< 0.001	< 0.001	0.004
Postoperative blood tests							
HB (g/dL)							
24 h	11.7 ± 1.1	12.2 ± 2.2	11.9 ± 0.8	0.430^a^	–	–	–
POD#3	11.2 ± 2.4	12.5 ± 3.4	11.6 ± 1.2	0.125^a^	–	–	–
Hct (%)							
24 h	33.5 ± 4.1	34.1 ± 3.9	33.7 ± 4.4	0.849^a^	–	–	–
POD#3	33.7 ± 2.9	34.6 ± 2.4	34.1 ± 3.1	0.466^a^	–	–	–
D-dimer (mg/L)							
24 h	9.6 ± 3.4	8.1 ± 2.9	8.4 ± 3.3	0.165^a^	–	–	–
POD#3	11.2 ± 4.1	9.8 ± 3.1	10.3 ± 3.8	0.335^a^	–	–	–
FIB (g/L)							
24 h	5.7 ± 2.1	5.1 ± 1.9	5.4 ± 2.3	0.546^a^	–	–	–
POD#3	7.4 ± 2.6	6.4 ± 1.3	6.8 ± 2.9	0.264	–	–	–
Real Hb decrease (g/dL)	1.5 ± 0.6	0.9 ± 0.3	1.2 ± 0.4	< 0.001^a^	< 0.001	0.026	0.002
Transfusion rate (%)	6.7%	0	0	0.129^b^	–	–	–

*Abbreviations*: *POD#3* the third postoperative day

*P*_*1*_ represents the P value obtained by comparison between control group and i.v. group;

*P*_*2*_ represents the *P* value obtained by comparison between control group and topical group;

*P*_*3*_ represents the *P* value obtained by comparison between i.v. group and topical group;

Intergroup comparisons performed using AVONA or Chi-square test (^a^ANOVA; ^b^Chi-square test)

### Postoperative blood tests

The results of the routine blood tests and blood coagulation tests in the three groups are summarized in Table 2. The mean values of postoperative Hb at 24 h in the control, i.v., and topical groups were 11.7 ± 1.1, 12.2 ± 2.2, and 11.9 ± 0.8 g/dL, respectively, with no significant difference between the groups (*P* = 0.430). The mean values of POD3 Hb in the control, i.v., and topical groups were 11.2 ± 2.4, 12.5 ± 3.4, and 11.6 ± 1.2 g/dL respectively, and there was also no significant difference between the groups (*P* = 0.125). Similarly, there were no significant differences in the mean Hct values between groups for the above two time points. However, there was a statistically significant difference in the average real Hb reduction among the three groups (*P* <  0.001): the values were 1.5 ± 0.6 g/dL in the control group, 0.9 ± 0.3 g/dL in the i.v. group, and 1.2 ± 0.4 g/dL in the topical group. The best effect was observed for the i.v. group (*P* = 0.002, versus the topical group).

The mean values of postoperative D-dimer after 24 h in the control, i.v., and topical groups were 9.6 ± 3.4, 8.1 ± 2.9, and 8.4 ± 3.3 mg/L, respectively, with no significant difference between groups (*P* = 0.165). The mean values of POD3 D-dimer in the control, i.v., and topical groups were 11.2 ± 4.1, 9.8 ± 3.1, 10.3 ± 3.8 mg/L, respectively, with no significant difference between groups (*P* = 0.335). Similarly, there were no significant differences for mean FIB values among the three groups at the above two time points (all *p* values > 0.05).

### Blood loss and transfusion

**Fig. 1:**
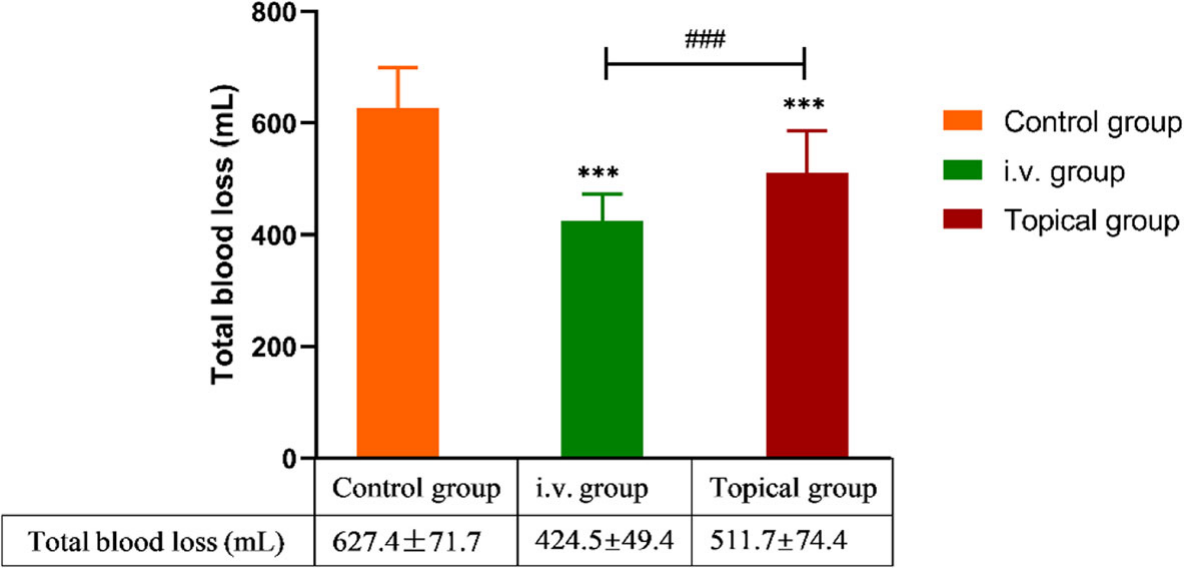
Comparison of TBL in the three groups. *** indicated *P* < 0.001 (i.v. group or topical group vs control group); ^###^ indicated *P* < 0.001 (i.v. group vs control group). Intergroup comparisons were performed using ANOVA

**Fig. 2:**
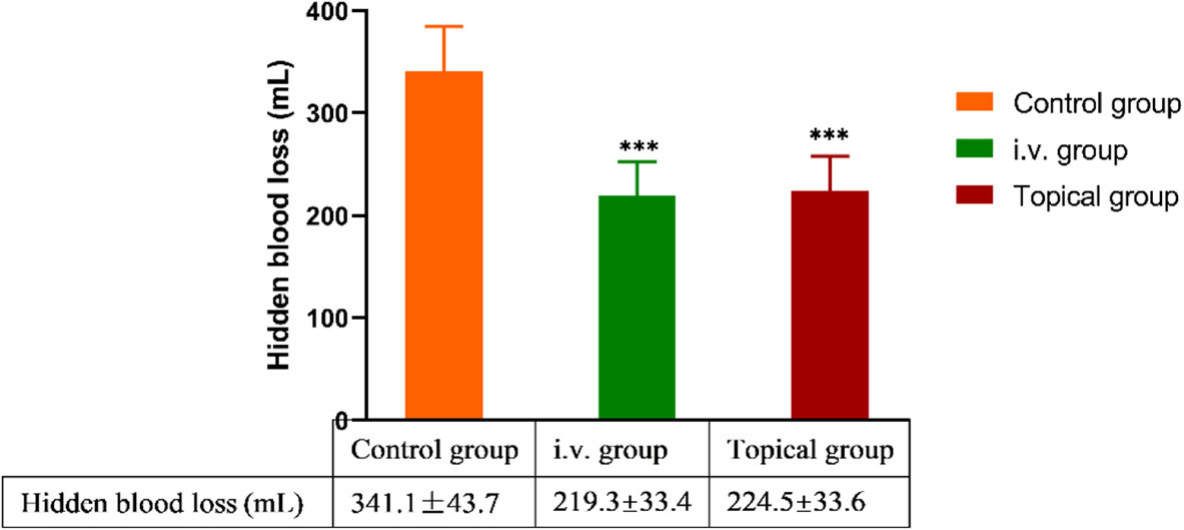
Comparison of HBL in the three groups. *** indicated *P* < 0.001 (i.v. group or topical group vs control group); there was no difference between the i.v. group and topical group. Intergroup comparisons were performed using ANOVA

After careful calculation and verification, we found that there were significant differences in TBL among the three groups (*P* <  0.001). The pairwise comparisons and statistical analyses between groups, namely, control group vs i.v. group, i.v. group vs topical group, and control group vs topical group, all showed statistically significant differences (Fig. 1). Similar results were also obtained for HBL; the HBL values in the control, i.v., and topical groups were 341.1 ± 43.7, 219.3 ± 33.4, and 224.5 ± 33.6 mL, respectively (all intergroup *P* values < 0.001) (Fig. 2).

The final analysis revealed that two patients in the control group were transfused with 2 units of PRBCs owing to symptoms of postoperative anemia, whereas no patients in the i.v. group and the topical group required blood transfusion. No statistical differences were found in the transfusion rate among the three groups (*P* = 0.129) (Table 2).

### Vascular events, wound complications, and adverse reactions

In this study, the venous plexus of the calf muscle was the most common site for DVT, followed by the popliteal vein. No severe complications, such as pulmonary embolism, myocardial infarction, or cerebral infarction, occurred to any patient enrolled in this study. There were no significant differences in wound complications and adverse reactions among the three groups. The specific data are described in Table 3.

**Table 3:** Vascular events, wound complications, and adverse reactions resulting from TXA

Variable	Control group (30 patients)	i.v. group (30 patients)	Topical group (30 patients)	*P* value (between groups)
Vascular events				
DVT	3	5	3	0.661
MI	0	0	0	–
CI	0	0	0	–
PE	0	0	0	–
GIH	0	1	0	0.364
Total	3	6	3	0.421
Wound complications				
Dehiscence	0	0	0	–
Hematoma	0	0	0	–
Edge necrosis	0	0	0	–
Infection	1	0	0	0.364
Total	1	0	0	0.364
Adverse reactions				
Epilepsy	0	0	0	–
Rash	0	1	0	0.364
Headache	2	4	1	0.338
Nausea and Vomiting	1	1	1	1.000
Total	3	6	2	0.260

*Abbreviations*: *TXA* tranexamic acid, *DVT* deep vein thrombosis, *MI* myocardial infarction, *CI* cerebral infarction, *PE* pulmonary embolism, *GIH* gastrointestinal hemorrhage

Chi-square test was performed for intra-group comparisons

### Pain, functional, and QoL assessment (Figs. 3, 4, 5)

**Fig. 3:**
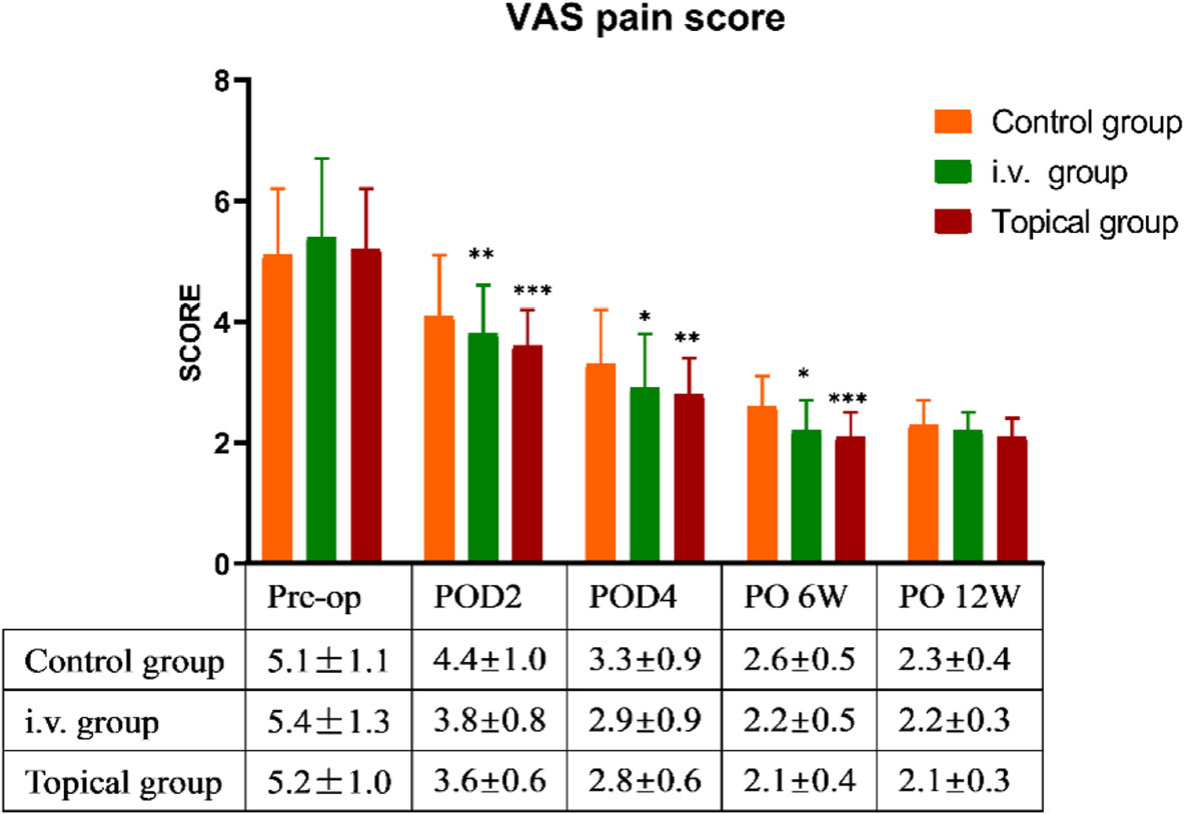
Comparison of VAS pain in the three groups. * indicated *P* < 0.05 (i.v. group or topical group vs control group); ** indicated *P* < 0.01 (i.v. group or topical group vs control group); *** indicated *P* < 0.001 (i.v. group or topical group vs control group); There was no difference between the i.v. group and topical group. Intergroup comparisons were performed using ANOVA. Abbreviations: two days after surgery, POD2; four days after surgery, POD4; six weeks after surgery, PO6W; twelve weeks after surgery, PO12W

According to our results, topical TXA had the best effect on pain control in the early postoperative period (POD2 and POD4, *P* = 0.002 and 0.025, respectively), as well as in the later periods (PO6W, *P* <  0.001).(Fig. 3).

**Fig. 4:**
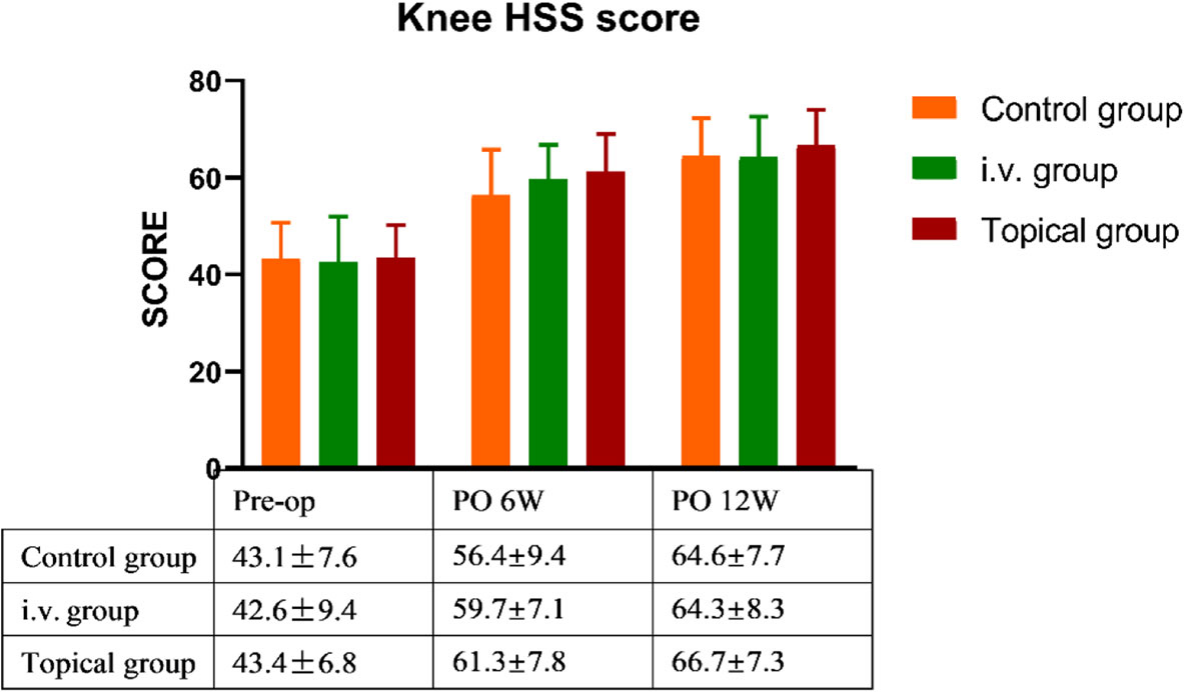
Comparison of knee function between the three groups. There was no difference among the groups at the above time points

**Fig. 5:**
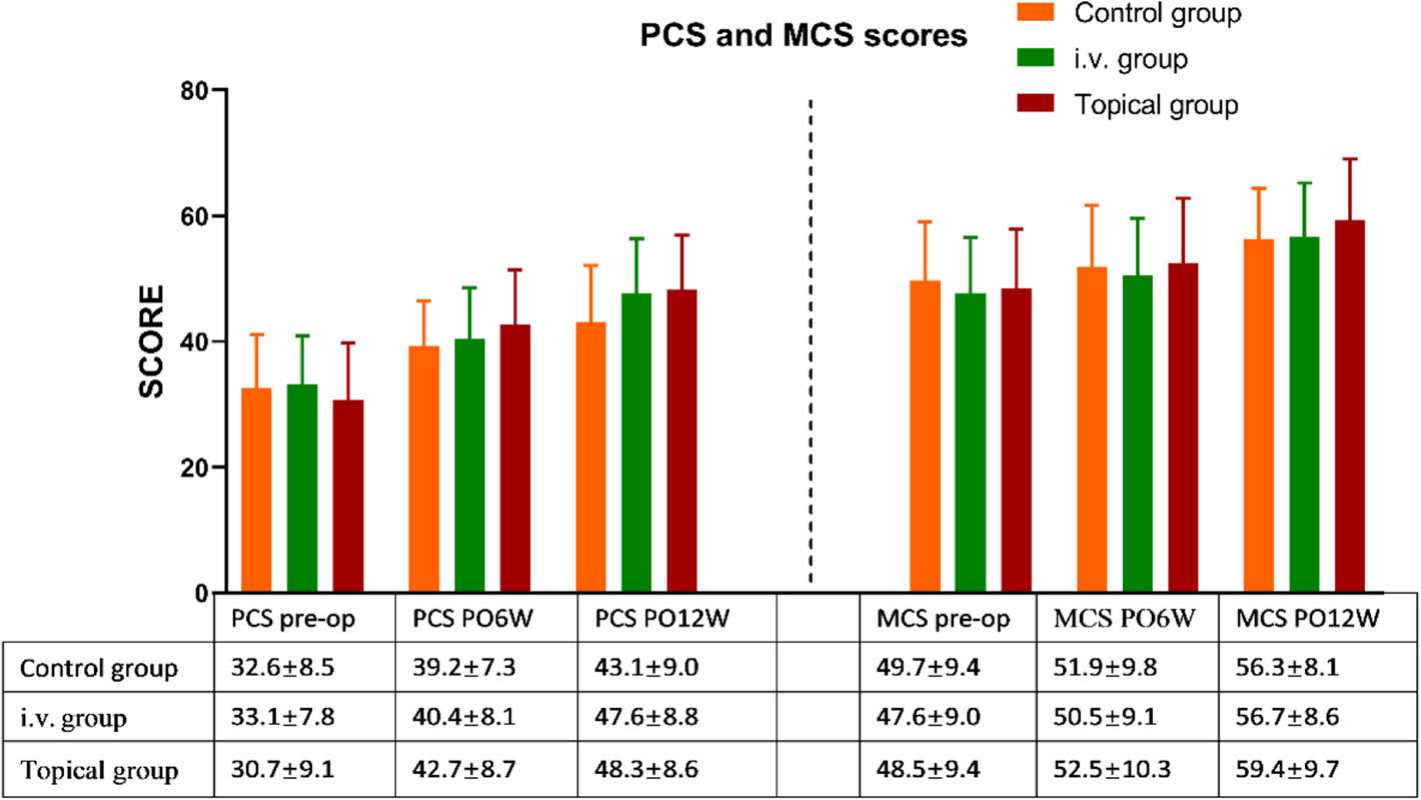
Comparison of PCS and MCS in the three groups. There was no difference among the groups at the above time points. Abbreviation: six weeks after surgery, PO6W; 12 weeks after surgery, PO12W

The knee function HSS scores were used to assess the knee function in patients both before and after surgery. Although there were no significant differences in postoperative follow-up among the three groups at PO 6 W and PO 12 W (*P* values: 0.052 and 0.432), it is worth mentioning that the topical group benefitted the most, followed by the i.v. group. (Fig. 4).

To assess the overall knee function, we used both the PCS and MCS of the SF-12. In PCS, the topical group showed a sustainable development advantage from the 6th week to the 12th week after surgery (*P* = 0.237 and 0.051, respectively). This advantage is likely to become more significant as the follow-up time increases. The same conclusion also applies to the comparison of MCS among the three groups. (Fig. 5).

## Discussion

Our research focus has always been on blood loss reduction during orthopedic surgery. Patients who have not developed anemia and have higher hemoglobin levels show better performance during rehabilitation training and may have additional benefits such as a better QoL [8, 30]. An effective blood preservation strategy is the key to ERAS after orthopedic surgery. TXA, an important component of blood management during orthopedic surgery, makes this strategy a reality [31–33].

In this study, the use of TXA reduced IBL and TPD. Compared to the control group, the i.v. regimen effectively reduced blood loss by approximately 28 mL (*P* <  0.001). The topical regimen can also significantly reduce TPD by approximately 92 mL compared to that in the control group (*P* <  0.001); this conclusion was also reached by Artit et al. [34] The i.v. regimen reduced real Hb reduction more effectively during the perioperative period, similar to the results of a study conducted by Tzatzairis et al. [35]

Sehat et al. [36] first proposed the concept of HBL and studied the HBL of 63 patients who underwent TKA. They found that HBL accounted for approximately 50% of the TBL. Guo et al. [37] found that the TBL of perioperative patients during hip replacement was approximately 859 mL and the HBL was approximately 525 mL, which accounted for 61% of the TBL. Foss et al. [38] studied the hidden blood loss after hip fracture and found that different surgical schemes caused a hidden blood loss of 500–1473 mL, which was approximately 2 to 3 times greater than the visible blood loss. Therefore, to reduce blood loss in orthopedic surgery, HBL reduction should be the priority. The main finding of our study was that both intravenous and topical TXA can effectively reduce TBL during the perioperative period, and that the intravenous regimen had the strongest effect (approximately 87 mL compared to the topical group, *P* <  0.001). Similarly, the perioperative HBL was also reduced; however, there was no significant difference between the i.v. regimen and topical regimen.

It has been proposed that the use of tourniquets will lead to excessive fibrinolysis and blood loss within the first 6 h after surgery [39]. In the current study, the second TXA application in the i.v. group was before the tourniquet release; thus, its effect may have been weakened since the fibrinolytic response was already in progress. The topical application of TXA allows it to rapidly reach the active bleeding point and directly interact with the wound, inhibiting the fibrinolytic reaction in the blood, promoting the formation of fibrin and maintaining a stable clot, reducing the leakage of blood to the surface of the damaged tissue, and exerting hemostatic effects [40]. Thus, this explains why there were no significant differences between the i.v. group and topical groups in IBL and TPD.

Thus far, most orthopedic clinical trials have been designed to test the hemostatic effect of TXA instead of its safety. In rare complications, such as pulmonary embolism, the current clinical trial sample size cannot reach a definitive conclusion. The results of this study showed that patients were safe whether they received i.v. or topical treatment. The i.v. regimen appeared to increase the incidence of vascular events and adverse reactions, but with fewer wound complications compared to the control group; however, this difference was not significant. Some studies have confirmed that plasmin not only promotes the activation of monocytes, platelets, and endothelial cells but also plays an important role in stimulating the release of inflammatory mediators and the induction of related pro-inflammatory gene expression. TXA is an inhibitor of plasmin; thus, ammonia TXA also has potential anti-inflammatory effects [41, 42]. In addition, reducing the perioperative blood transfusion rate may also reduce the incidence of wound complications [43]. The potential mechanism and advantage of the topical administration of TXA is to directly target the site of bleeding just before wound closure. The inhibition of local fibrinolytic activity helps prevent fibrin clot dissolution and increases its volume and strength at the raw surgical surfaces, enhancing microvascular hemostasis [44]. In addition, TXA has lower systemic absorption. At present, topical articular cavity administration has become one of the best alternatives compared with i.v [15, 16, 18–20, 41].. The results of our study also confirmed this result, since the topical group had a lower incidence of vascular events and wound complications, and also provided the benefit of reducing blood loss. Hence, we believe that the use of topical TXA in this study has been shown to be safe. The Chinese guidelines for the prevention of venous thromboembolism after orthopedic surgery recommend that anticoagulants should be used for at least 10 to 14 days after orthopedic surgery; however, as surgeons, we must weigh the expected benefits and risks, and determine the balance between the coagulation and fibrinolysis systems. In our institution, we used TXA to achieve this balance and have presented our preliminary results in this study [14, 29].

Another important finding in this study was that pain was significantly reduced in patients in the topical group during the early and late postoperative periods. Patients in the topical group received 75 mL TXA, which was retrogradely perfused into the deep part of the incision through the drainage device. The drainage tube was temporarily clamped for 4 h and a self-adhesive elastic bandage was used to compress the incision again. We believe that the intra-articular pressure in patients in the topical group was higher than that in the control and i.v. groups, which helped reduce pain caused by hematoma stimulation and swelling [45]. In addition, the HSS of patients in the topical group had a slight advantage in postoperative knee function. This advantage is reflected in the fact that patients can perform a wider range of knee mobility exercises and early ambulation. This can explain the long-term, higher PCS in the topical group in terms of postoperative QoL; however, this difference was not adequate to show statistical significance.

There are some limitations to our study. First, the sample size of this study was small and the results were obtained from a single center. A large-scale prospective, randomized case-control study is needed to further confirm these results. Second, according to the Chinese guidelines for the prevention of venous thromboembolism after orthopedic surgery, all patients received preventive anticoagulation after admission, which may have affected the rate of blood loss. Third, blood loss in postoperative wound dressings was not measured in this study.

## Conclusion

In summary, for complex Schatzker type V and VI tibial plateau fractures, the use of TXA is reasonable, safe, and effective. The i.v. regimen offers the advantage of lower perioperative blood loss, whereas the topical regimen can reduce the incidence of vascular events, wound complications, adverse reactions, as well as reduce postoperative pain.

## Abbreviations

TXA: Tranexamic acid
i.v.: Intravenous
QoL: Quality of life
TBL: Total blood loss
HBL: Hidden blood loss
RBC: Red blood cell
DVT: Deep vein thrombosis
MAP: Mean arterial blood pressure
ASA: American Society of Anesthesiologists score
Hb: Hemoglobin
Hct: Hematocrit
FIB: Fibrinogen
BMI: Body mass index
HSS: Hospital for special surgery
SF-12: The 12-item Short Form Health Survey
VAS: Visual analog scale
PE: Pulmonary embolism
IBL: Intraoperative blood loss
TPD: Total postoperative drainage
PBV: Preoperative blood volume
PCS: Physical component summary
MCS: Mental component summary
